# Characteristics and Pathogenicity of the Cell-Adapted Attenuated Porcine Epidemic Diarrhea Virus of the Non-S INDEL Cluster

**DOI:** 10.3390/pathogens10111479

**Published:** 2021-11-13

**Authors:** Thi Thu Hang Vu, Minjoo Yeom, Hyoungjoon Moon, Thi Nhan Tran, Van Phan Le, Daesub Song

**Affiliations:** 1 College of Pharmacy, Korea University, Sejong 30019, Korea; vutth@korea.ac.kr (T.T.H.V.); virus1122@korea.ac.kr (M.Y.); 2 College of Healthcare & Biotechnology, Semyung University, Jecheon 27136, Korea; mhj1219@semyung.ac.kr; 3 Research Unit, Green Cross Veterinary Products, Yongin 17066, Korea; 4 R&D laboratory, AVAC Vietnam Company Limited, Hung Yen 163530, Vietnam; nhan.victory@gmail.com; 5 College of Veterinary Medicine, Vietnam National University of Agriculture, Hanoi 131001, Vietnam

**Keywords:** porcine epidemic diarrhea, attenuated vaccine, pathogenicity, LLC-MK2, genetic variations

## Abstract

The high antigenic diversity of porcine epidemic diarrhea virus (PEDV) means that porcine epidemic diarrhea (PED) is a challenge for the global pig industry. Understanding the circulation of the virus to determine an optimal vaccine strategy is important in controlling the disease. In this study, we describe the genetic diversity of circulating PEDV based on the full sequences of spike genes of eight positive samples collected in Vietnam since 2018. Additionally, we developed a live attenuated vaccine candidate from the cell-adapted PEDV2 strain, which was continuously passaged until level 103 in VERO-CCL81 cells. PEDV2-p103, which belongs to the emerging non-S INDEL cluster, exhibited low virus shedding, did not induce lesions in the small intestine of challenged piglets, and had a high titer in the VERO-CCL81 cell at 48 h post-infection. These results suggest that the PEDV2-p103 strain could be a potential oral attenuated vaccine, and its immunogenicity and efficacy should be further assessed through in vivo tests.

## 1. Introduction

Porcine epidemic diarrhea (PED) is an acute and highly contagious diarrheal disease caused by the PED virus (PEDV) in pigs of any age. Clinical symptoms of PED include diarrhea, vomiting, anorexia, dehydration, and weight loss [1]. PED is currently one of the most feared diseases in the global pig industry due to (i) its rapid transmission from pig-to-pig and farm-to-farm (through fecal–oral routes) [2]; (ii) its high mortality rate in piglets (80–100%) [3]; and (iii) the lack of effective treatments or vaccines for emerging highly virulent PEDV strains [4]. PEDV is an enveloped, positive-sense RNA virus that belongs to the family *Coronaviridae*, genus *Alphacoronavirus*. The genomic RNA of PEDV is approximately 28 kb, with a 5′cap and a 3′poly A tail comprising seven open reading frames (ORFs) encoding non-structural proteins and four structural proteins [1,5,6,7,8,9]. ORF1a, ORF1b, and ORF3 encode non-structural proteins, of which ORF3 is reported to be involved in the regulation of viral replication and virulence [10,11,12]. The four structural proteins are the spike (S), envelope (E), membrane (M), and nucleocapsid (N) proteins. The S protein is a glycoprotein on the surface of the virus that plays a critical role in the interaction of the virus with specific host cell receptors and in viral pathogenicity. The S protein can be cleaved into two subunits: S1, which contains the main receptor binding sites and neutralizing epitopes; and S2, which contains a fusion peptide and a transmembrane domain involved in the entrance of the virus to the host cell [13,14]. The diversity of the spike genes determines the genetic diversity among PEDV strains; therefore, understanding this is necessary for the development of PED vaccines [3,15,16]. 

There have been three milestones in the global spread of PEDV. First, since its first detection in England in 1971, PEDV strains from the classical group have been collected and isolated. During the period until 2010, PEDV was transmitted from Europe to Asia and caused epidemics in China, Korea, Japan, Taiwan, Philippines, Thailand, and Vietnam [17,18,19,20,21]. Second, from 2010, PEDV strains with higher pathogenicity than previous strains re-emerged in China and other Asian countries, causing many outbreaks with devastating effects in the swine industry. These PEDV strains have been classified into the Asian (ASI) non-S INDEL (insertions and deletions in spike gene) cluster, or 2a group. Finally, in April 2013, PEDV was first detected in the United States. These PEDV strains were classified into the North American (NA) non-S INDEL cluster, or 2b group. Following the outbreaks of NA non-S INDEL variants, novel variants have emerged with insertions and deletions in the spike proteins that are less pathogenic than non-S INDEL variants [2]. These variants were classified into S INDEL or group 1b. The emergence of non-S INDEL and S INDEL variants from 2010 reduced the efficacy of available vaccines that were derived from the classical strains [4]. 

In this study, we report on the genetic diversity of PEDV strains that circulated in Vietnam in 2018 to highlight the need for new vaccines that are effective for the prevention of emerging and re-emerging PEDV strains. Additionally, we present an attenuated emerging non-S INDEL PEDV strain that could serve as a second-generation attenuated PEDV vaccine candidate.

## 2. Results

### 2.1. Genetic Variations of PEDV in Field Isolates from Vietnam in 2018

**Figure 1:**
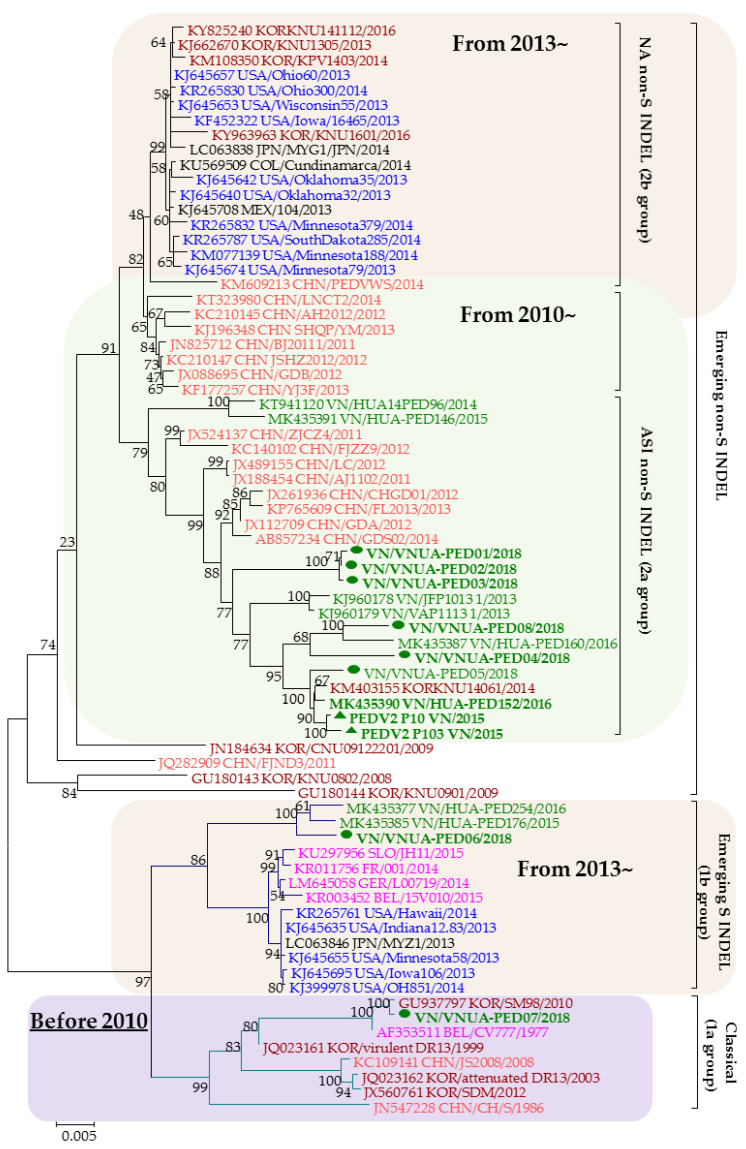
Phylogenetic tree of spike genes of the PEDV strains. The number on each branch indicates the bootstrap value. The scale represents the nucleotide substitutions per site. Vietnamese PEDV strains in this study were labeled using green bold letters with a green circle (eight of the PEDV strains in this study) or a triangle (PEDV2-p10 and PEDV2-p103 strains). Each reference PEDV strain is indicated in the format: GenBank accession number/Country (letter codes: BEL, Belgium; CHN, China; COL, Colombia; GER, Germany; JPN, Japan; KOR, Korea; MEX, Mexico; USA, the United States; VN, Vietnam)/strain name/year of sample collection. Vietnamese, U.S., Chinese, European, and Korean PEDV strains are in green, blue, orange, purple, and garnet, respectively.

The phylogenetic tree was constructed based on the full spike genes of eight PEDV strains collected in Vietnam in 2018, two passages of PEDV2 strains isolated in Vietnam in 2015 (PEDV2-p10 and PEDV2-p103), and reference strains (Figure 1). The phylogenetic tree revealed the genetic relationship of PEDV strains at different time points. Most current circulating PEDV strains belong to the 2a group.

Several lines of evidence support a theory regarding the origin of the PED virus through the recombination of related coronaviruses [2]. Previous investigations have indicated that the most frequent mutations in PEDV strains are found in the receptor-binding domain of the S protein [3,15,16]. An analysis of the spike gene sequences of PEDV strains collected in Vietnam in 2018 revealed that 6/8 strains could be classified as emerging ASI non-S INDEL sequences; one strain belonged to the S INDEL cluster; and another strain had a spike gene closely related to the KOR/SM98/2010 strain, which belongs to the classical group. Deletions and insertions in the spike gene of these strains mainly appeared in the S1 subunit, which is responsible for receptor binding. Mutations in this domain can affect the binding capacity of host cells. The strains used in this study, PEDV2-p10 and PEDV2-p103, belonged to the ASI non-S INDEL cluster, or 2a group.

Four neutralizing epitopes of the PEDV S protein have been identified including the CO-26K-equivalent (COE) domain (amino acid 499-638), SS2 (amino acid 748–755), SS6 (amino acid 764–771), and 2C10 (amino acid 1368–1374), which are based on S protein of the PEDV CV777 strain [22,23,24]. Two of the four epitopes, SS2 and 2C10, were conserved [25]. They were also conserved in all isolates in this study (data not shown). Therefore, in this study, we compared the neutralizing epitopes, COE and SS6, of our Vietnamese strains to reference PEDV strains from different genotypes (Table 1). Notably, the VN/PEDV07/2018 strain presented the epitope COE, and the SS6 regions were the same as those of the CV777 strain, despite this strain having the closest relationship in the phylogenetic tree with the SM98 strain. Additionally, SM98 was identical to CV777 in COE but not SS6 (navy rectangle in the table). Three positions, A517, T549, and G594 (blue rectangles in the table), exhibited differences among the genotype groups, and were substituted with serine in emerging non-S INDEL and S INDEL strains compared with the classical strains. However, in this study, the Vietnamese strains in the emerging non-S INDEL group had no serine substitution at A517, although 4/6 strains exhibited a serine substitution at T549. PEDV2 passages (marked with a green rectangle in the table) belonged to the same 2a group, but they only contained serine substitution at G594.

### 2.2. Isolation and Characterization of the PEDV2 Passages

The PEDV2 strain was cultured in VERO CCL-81 cells. PEDV2-p10, which had spike gene and ORF3 gene sequences identical to PEDV2-p0, was multiplied in a large volume and stored as a representative of the original seed for the subsequent experiments. The PEDV2 strain was continuously passaged in VERO-CCL81 cells until passage 100. Each passage lasted 48–72 h, until a 90–100% cytopathic effect (CPE) was observed. At passage 100, extended deletions in the ORF3 gene were found. PEDV2 virus particles from passage 100 were purified by the plaque assay. A total of 50 plaques collected had identical spike and ORF3 gene sequences. A PEDV2-p101.1 clone was chosen for the subsequent experiments, and PEDV2-p103.1 was stored in a large volume as an attenuated candidate for the next experiments. The strain referred to as PEDV2-p103 in this study was the PEDV2-p103.1 clone. 

In the spike gene of the PEDV2-p103 strain, ten single-nucleotide mutations and a six-nucleotide deletion resulted in nine amino acid mutations and two amino acid deletions in the S protein compared with the PEDV2-p10 S protein (Table 2). Among them, 6/9 mutations and deletions were in the S1 subunit, and 3/9 mutations were in the S2 subunit. Notably, the double amino acid deletion was repeated in an independent passage of PEDV2 in VERO-CCL81 cells (2P/PEDV-p21) but did not occur in any of the passages in LLC-MK2 cells (LLC-MK2/PEDV2-p50).

In the ORF3 gene of PEDV2, a 38-nucleotide deletion was found in passage 26 (Table 3). This deletion extended to six additional nucleotides at the 3′ end at passage 103. Both deletions caused truncation of the ORF3 protein at amino acid position 195. The rectangles in Table 3 represent the codon change resulting from nucleotide deletions. No deletion was observed until passage 50 in the passage process of LLC-MK2 cells.

### 2.3. Selection of Permissive Cell Lines with PEDV2

Epithelial cell lines isolated from monkey kidneys have been extensively used for research on coronaviruses. These cell lines include Vero E6 for severe acute respiratory syndrome coronavirus (SARS-CoV), Middle East respiratory syndrome coronavirus (MERS-CoV), and SARS-CoV-2; VERO-CCL81 for PEDV; and LLC-MK2 for human coronavirus NL63 [26,27,28]. We selected the cell lines VERO-CCL81, LLC-MK2, and MARC-145, a cell line derived from monkey kidney epithelial cells, for the following experiments. In addition, the pig-derived cell lines ST, IPEC-J2, and PK15 were selected. ST and IPEC-J2 were reported to express the aminopeptidase N receptor, one of the PEDV receptors, on the cellular surface; and PK15 is an epithelial cell line derived from porcine kidney.

The permissiveness to PEDV2 strains (passages 10 and 103) of these six cell lines was tested. The ΔCt between day 0 and day 4 varied from negative to 14.78 (MARC-145) and 17.23 (VERO-CCL81) for PEDV2-p10 and PEDV2-p103, respectively. The TCID_50_ values revealed that LLC-MK2 and VERO-CCL81 were more permissive of PEDV at both passage levels than the other cell lines tested. The three cell lines originating from monkey kidney epithelial cells were more permissive than the porcine-originated cell lines, except for ST cells, and produced CPE at 40 h (LLC-MK2, VERO-CCL81) and 48 h (MARC-145) post-infection (Table 4). Among the porcine cell lines, ST was the most permissive for proliferating the PEDV2 virus.

**Figure 2:**
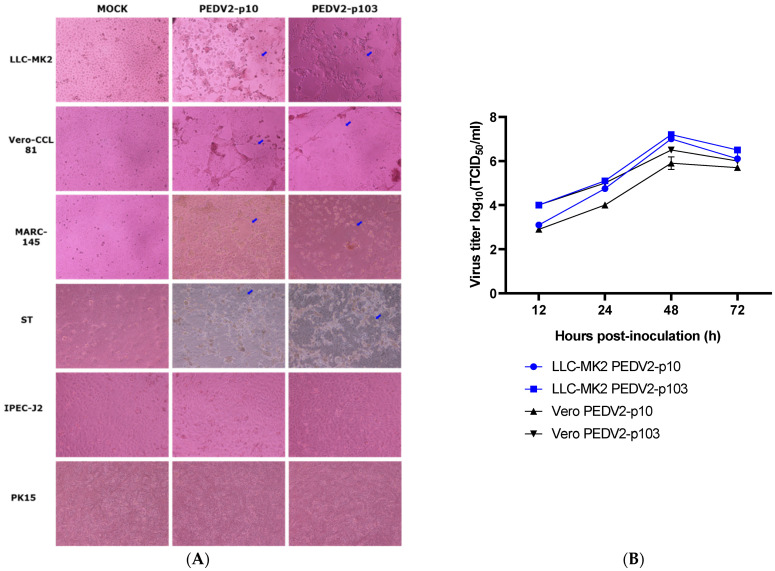
The infectivity of PEDV2 passages 10 and 103 in the tested cell lines. (**A**) Morphological changes observed in the different cell lines (×1000) at 48 h post-infection; (**B**) Growth curve of PEDV2 strains in Vero (VERO-CCL81) and LLC-MK2 cell lines. Blue arrows indicate the CPE of PEDV2.

The morphological differences observed in the six cell lines are shown in Figure 2A. The CPEs were clearly observed in LLC-MK2, VERO-CCL81, MARC-145, and ST cells in the presence of 5 µg/mL trypsin in the infectious medium at 48 h post-infection. The growth curves of PEDV2-p10 and p103 on LLC-MK2 and VERO-CCL81 cells were characterized (Figure 2B). The viral titer peaked at 48 h post-infection in both virus strains and cell lines. The permissiveness of LLC-MK2 and VERO-CCL81 to PEDV2-p10 and p103 was similar.

### 2.4. Pathogenicity of PEDV2 Passages in Neonatal Piglets

**Figure 3:**
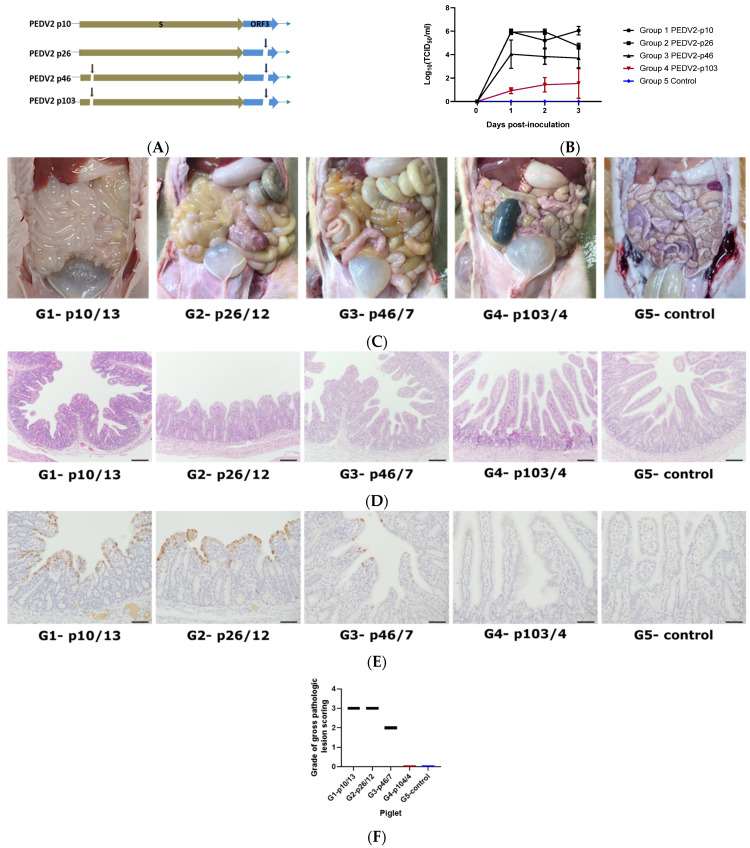
PEDV detection in the piglets after inoculation with the different passages of PEDV. (**A**) Description of the genetic changes in PEDV2 passage levels in *spike* and ORF3 genes; (**B**) Viral shedding of PEDV in piglet feces post-infection with MOCK/different passages of PEDV2—statistical significance was assessed using two-way analysis of variance (ANOVA), *p*-value < 0.001; (**C**) Post-mortem signs in piglet intestines after inoculation with different PEDV2 passages. Black arrows indicate the pathogenic signs in the small intestines; (**D**) Hematoxylin and eosin-stained tissue sections of small intestines after inoculation with different PEDV2 passages (bar = 100 µm); (**E**) Detection of PEDV antigens by immunohistochemistry (IHC) in small intestinal tissues after inoculation with different PEDV2 passages (bar = 50 µm). Black arrows refer to positive antigens; (**F**) Grades of gross pathologic lesion scoring of representative piglets of each group, 0: normal, 1: mild villous atrophy, 2: moderate villous atrophy, 3: severe villous atrophy; G1-p10/13, piglet number 13 of group 1 infected with PEDV2-p10; G2-p26/12, piglet number 12 of group 2 infected with PEDV2-p26; G3-p46/7, piglet number 7 of group 3 infected with PEDV2; piglet number 4 of group 4 infected with PEDV2-p103; G5-control, piglet of group 5, negative control.

In the pathogenicity test, piglets were divided into five groups: four groups were inoculated with four different PEDV2 passage levels (PEDV2-p10, p26, p46, and p103), and the negative control group was inoculated with PBS. The four virus samples of different passage levels were genetically different. Passage 26 had a 38-nucleotide deletion in ORF3; passage 46 had the same deletion in ORF3 and a significant 6-nucleotide deletion in spike gene; and passage 103 had a 44-nucleotide deletion in ORF3 and a 6-nucleotide deletion in spike gene (Figure 3A).

In group 1 (PEDV2-p10) and group 2 (PEDV2-p26), piglets started to have watery diarrhea from day 1 and lost 20% of body weight from day 2 post-inoculation; in group 3 (PEDV2-p46), piglets started to have sticky feces from day 2 post-inoculation, whereas in group 4 (PEDV2-p103) and group 5 (non-inoculated), the piglets were healthy. Although virus shedding in the four inoculated groups kept increasing, significantly lower virus shedding was found in group 4 relative to groups 1, 2, and 3 for three days post-inoculation (Figure 3B). On day 3 post-inoculation, the mean virus shedding in group 4 piglets was 10^4.51^-fold lower than that in the group 1 piglets. Previous studies have shown that viral shedding titers are highest from days 3 to 4 post-challenge, and on these days, pathogenic signs were the clearest [29,30,31,32]. On day 4 post-inoculation, piglets from each group were necropsied to observe the lesions in the small intestines (Figure 3C). Small intestines from piglets in group 4 and group 5 were normal with no pathogenic signs, whereas the small intestine of groups 1, 2, and 3 piglets displayed typically pathogenic signs (indicated with black arrows) such as fluidic and distended walls of the small intestine. The most severe lesions were found in group 1. Histopathological analysis revealed the degeneration and atrophy of intestinal villi and a high density of the virus on the intestinal villi of group 1 and group 2 piglets (Figure 3D–F). The density of virus was lower in the intestinal villi of group 3 piglets compared to those in groups 1 and 2. No virus was detected in the intestinal villi of group 4 or group 5 piglets. Antigen positive signals are depicted in brown in Figure 3E.

## 3. Discussion

PEDV was first reported in the south of Vietnam in 2009 [17]. From 2012 to 2016, PEDV strains belonging to the four classification groups circulated in many regions of Vietnam [33,34]. In this study, eight PEDV strains collected in Vietnam in 2018 were classified into three groups. Of the eight strains, six belonged to the emerging ASI non-S INDEL group, only one strain belonged to the S INDEL group, and one strain belonged to the classical group. Although the number of strains in this study was small, the prevalence of emerging PEDV non-S INDEL strains was in line with that reported in previous studies in China and Japan [35,36]. In recent studies, 23/25 PEDV strains collected from the north of Vietnam during 2012–2015 and 28/30 PEDV strains during 2015–2016 collected in Vietnam (north, middle, and south) belonged to the non-S INDEL cluster [33,34]. Our findings illustrate the diversity of PEDV circulating in Vietnam and are consistent with those of previous reports. Additionally, our results highlight the need for novel vaccines or multiple-variant vaccines for the effective prevention of PED.

Several PEDV vaccines have been developed due to the high prevalence of severe PED outbreaks. Attenuated vaccines, which are highly immunogenic and induce long-lasting immune responses, are commonly used in Asian countries. The first attenuated vaccine was developed in 1994 and was based on the cell-adapted CV777 strain [37]. Subsequently, the live attenuated P-5V vaccine was approved in Japan in 1997 [38]. Three other attenuated vaccines have been released in Korea based on cell-adapted strains: KPEDV-9, SM98, and DR-13. Among them, the oral administration of DR-13 was reported to be the most efficient option. This vaccine was also registered in the Philippines in 2011 [19,20,30,39]. These attenuated vaccines were produced from classical strains (group 1a) and have become less protective against highly pathogenic strains that have appeared since 2013. Since 2015, live attenuated bivalent or trivalent vaccines (combined with transmissible gastroenteritis virus (TGEV) or TGEV and porcine rotavirus, respectively) based on the PEDV strain of the 2a group have been used in China [40]. From 2020, an oral attenuated vaccine from the KNU-141113S-DEL5/ORF3 strain (of 2b group) has been administered to sows in Korea [41]. Due to the diversity of PEDV, it is necessary to create vaccines from newly emerging strains. In this study, PEDV2-p46 and PEDV2-p103 had a signature double amino acid deletion in the S protein and a truncated ORF3 protein, whereas PEDV2-p103 had a six-nucleotide deletion in the ORF3 gene. Recent studies have reported that viruses with deletions in ORF3 or truncated ORF3 are not pathogenic in piglets [29,42]. However, our results show that only the pathogenicity of PEDV2-p103 was attenuated. PEDV2-p10 and PEDV2-p103 were classified into the ASI non-S INDEL cluster or 2a group (Figure 1). Mutations in the S protein of PEDV2-p103 were not located in the neutralizing epitope regions; therefore, this did not lead to reduced immunogenicity. Importantly, PEDV2-p103 presented a high titer (>10^7^ TCID_50_/mL) in VERO-CCL81 cells.

In a previous study, we succeeded in creating an oral live attenuated vaccine from the PEDV DR13 strain [30]. This vaccine was effective in reducing mortality in suckling piglets and is used widely in Asia [4]. Following the same procedure, our goal was to create a new effective oral attenuated vaccine effective against the emerging PEDV2 strain. PEDV2-p103 caused: (i) low fecal virus shedding; (ii) no pathogenic signs in the piglets; and (iii) negative histopathology in intestine villi. Hence, to establish a novel attenuated vaccine, the immunogenicity in pregnant sows and the protective efficacy in the respective piglets of the PEDV2-p103 strain should be assessed.

In this study, PEDV2 showed diverse mutation patterns when it was passaged in VERO-CCL81 and LLC-MK2 cells. This result suggested that the cell adaptation of the PEDV2 virus varied in different cell lines. The distinct mutations profile in the viral genome might have caused the different viral characteristics observed. For example, it might explain the decrease in PEDV2-p103 titers observed in MARC-145 cells, but not VERO-CCL81 or LLC-MK2 cell lines compared to that of PEDV2-p10. Further experiments are required to clarify these behaviors.

Herein, we report the potential of LLC-MK2 for the proliferation of PEDV. Porcine cell lines were less permissive to PEDV2 than monkey kidney epithelial cells, although the expression of APN receptors has been reported in ST and IPEC-J2 cells. This suggests that a receptor other than APN may be associated with the viral entry [43,44]. The three cell lines from monkey kidney epithelial analyzed were more permissive to PEDV2 than the porcine cell lines, and the porcine kidney epithelial cell line PK15 was not susceptible to PEDV2. The mechanism underlying the distinct prevalence is unknown and needs to be elucidated in the future.

## 4. Materials and Methods

### 4.1. Virus and Cell Lines

The PEDV2 strain was isolated from a farm in Vietnam in 2015. Eight samples positive for PEDV were collected in Vietnam in 2018.

Three monkey kidney cell lines (LLC-MK2, VERO-CCL81, and MARC-145) and three porcine cell lines (IPEC-J2, ST, and PK15) were utilized in this study. These cell lines were stored and cultured in our laboratory. All cells were negative for *Mycoplasma* contamination. The cells were cultured in Dulbecco’s modified Eagle’s medium (DMEM, Corning^®^, Wujiang, Jiangsu, China) containing 1% antibiotic (Hyclone^™^, Logan, UT, USA) and 5% fetal bovine serum (FBS, AusgeneX, QLD, Australia).

### 4.2. RNA Extraction and Quantitative RT-PCR

RNA from 200 µL of culture supernatant or fecal swab was isolated with the QIAamp Viral RNA Mini Kit (QIAGEN, Hilden, Germany) and eluted in 50 µL. PEDV RNA was quantified using a Light Cycler 96 system (Roche, Indianapolis, IN, USA) with SensiFAST Probe No-ROX One-Step Kit (Bioline, Camarillo, TN, USA) using 20 µL of isolated RNA, 2× buffer, 10 µM forward primer (5′-CGCAAAGACTGAACCCA CTAATTT-3′), 10 µM reverse primer (5′-TTGCCTCTGTTGTTACTTGGAGAT-3′), and a 10 µM probe (5′-FAM-TGTTGCCATTGCCACGACTCCTGC-BHQ1-3′) [45]. The amplification steps were as follows: 10 min at 45 °C, 2 min at 95 °C, and 45 cycles of 15 s at 95 °C and 20 s at 60 °C.

### 4.3. Sequencing and Phylogenetic Analysis

The spike gene and the ORF3 gene were amplified using a Veriti^®^ 96-Well Thermal Cycler (Applied Biosystems^®^, Singapore) with SuperScript^®^ III One-step RT-PCR with Platinum^®^ *Taq* Kit (Invitrogen^™^, Waltham, CA, USA) from the extracted RNA of PEDV samples. Primers for spike gene sequencing were designed according to the alignment of the spike genes of the PEDV strains collected in Vietnam in 2012–2016 from GenBank. The primers used for sequencing in this study are listed in Table 5. Briefly, 3 µL of RNA was added to a reaction mixture containing 12.5 µL of buffer, 1 µL of each specific primer (10 µM), and 1 µL of enzyme mix, and diethyl pyrocarbonate (DEPC)-treated deionized water (DW) was used to achieve a final reaction volume of 25 µL. The cycling conditions were as follows: 55 °C for 30 min, 95 °C for 2 min, followed by 40 cycles of 95 °C for 30 s, 55 °C for 30 s, 68 °C for 1 min or 40 s (S fragments or ORF3, respectively), and a final extension step at 68 °C for 10 min. Samples were stored at 4 °C. The RT-PCR products were visualized by electrophoresis on a 1% agarose gel containing RedSafe^™^ Nucleic Acid Staining Solution (iNtRON, Gyeonggi-do, Korea). Correct bands were excised and purified using a QIAquick Gel Extraction Kit (QIAGEN, Hilden, Germany), according to the manufacturer’s instructions. All of the purified products were sequenced by Bionics Co. Ltd., Daejeon, Korea).

### 4.4. Virus Infection and Titration

The PEDV2 virus was proliferated in VERO-CCL81 cells, as described previously, with some modifications [18,48]. The cells were seeded in flasks or plates one day before infection. On the infection day, the monolayer cells were washed once with 1× phosphate-buffered saline (PBS) prior to inoculation with PEDV or diluted PEDV for 1 h. Thereafter, the cells were washed once with 1× PBS and then treated with PEDV infectious medium, containing DMEM with 1% antibiotic, 0.3% Bacto^™^ Tryptose Phosphate Broth (BD Difco^™^, Franklin Lakes, NJ, USA), 0.02% yeast extract (Gibco^®^, Grand Island, NY, USA), and 5 µg/mL trypsin (Corning^®^, Corning, VA, USA). After 48–72 h, PEDV was harvested by thawing the flasks/plates and centrifuging them at 4000 rpm at 4 °C for 20 min. The supernatant was then collected and stored in a deep freezer. The mean tissue culture infectious dose (TCID_50_) was calculated using the Spearman–Kärber method [49,50].

### 4.5. Isolation and Attenuation of the PEDV2 Virus in VERO-CCL81 Cells

Small intestine samples collected from infected piglets were homogenized. After centrifugation, the supernatant was filtered through a 0.2 µm syringe filter and was used to infect VERO-CCL81 cells for 1 h. Thereafter, the viral inoculum was removed, PEDV infectious medium was added, and the infected cells were cultured in an incubator (37 °C, 5% CO_2_). CPEs could be observed from the third passage. The PEDV2 isolate was passaged continuously until level 103 in VERO-CCL81 cells. Every 10 passages, virus samples were sequenced and titrated.

### 4.6. Virus Purification

The cell monolayer was incubated with PEDV at a ten-fold serial dilution ranging from 10^−1^ to 10^−5^ for 1 h. Infected cell monolayers were washed three times with 1× PBS and covered with 3 mL of 0.8% Bacto^™^ agar (BD Difco^™^, Franklin Lakes, NJ, USA) in 1× Minimum Essential Media (MEM (10×), Gibco^®^, Grand Island, NY, USA) containing 1% antibiotic, 0.3% Bacto^™^ Tryptose Phosphate Broth, 0.02% yeast extract, and 5 µg/mL trypsin. After 72 h, plaques were observed by staining with 0.01% neutral red for 2 h. Single plaques were picked and cultured for screening. Plaques were sequenced and the titer was estimated. The plaque with the highest titer was then selected for the seed.

### 4.7. Culture of PEDV2 in Different Laboratory Cell Lines

Cell lines were seeded in 48-well plates at a density of 4 × 10^4^ cells/well in growth medium until they reached 80–90% confluence. Then, the cell lines were inoculated with PEDV2-p10 and PEDV2-p103 at a multiplicity of infection of 0.01. Following the addition of the infectious medium, the cells were observed for specific CPEs. Supernatants were collected at 0 h and 96 h post-infection, and the viral titer was quantified using qRT-PCR and the TCID_50_ assay.

### 4.8. Pathogenicity of PEDV2 Passages in Neonatal Piglets

PEDV2 strains at four passage levels (PEDV2-p10, PEDV2-p26, PEDV2-p46, and PEDV2-p103) were selected for pathogenesis studies in piglets. Twenty piglets were housed in separate cages and randomly allocated to five groups. At five days of age, piglets in each group were orally inoculated with 1 mL of one passage level of PEDV2 and PBS (negative control). The dose of the viral inoculum was 10^5^ TCID_50_ in 1 mL volume per piglet. Clinical symptoms were recorded until day 4 post-inoculation. Fecal swabs were collected daily to evaluate viral shedding. Necropsy was conducted on day 4.

### 4.9. Histopathology and Immunohistochemistry (IHC)

Small intestinal tissue samples of each piglet were collected and fixed with 10% formaldehyde. Fixed samples were sent to OPTIPHARM Co. Ltd., Chungcheongbuk-do, Korea, for histopathology and IHC.

### 4.10. Bioinformatic Analysis

Nucleotide and deduced amino acid sequences were aligned using BioEdit v7.2.6.1. A maximum likelihood phylogenetic tree was constructed using the general time reversible nucleotide substitution method with a bootstrap test of 1000 replicates in MEGA 6.06 software [51]. Results are expressed as the mean ± standard deviation (SD) and were evaluated using GraphPad Prism v.8.4.3 (GraphPad Software, San Diego, CA, USA).

## 5. Conclusions

In this study, we have reported the genetic diversity of PEDV circulating in Vietnam in 2018 to highlight the need for new vaccines, which are effective for the prevention of emerging and re-emerging PEDV strains. In the preliminary in vivo test, the highly cell passaged PEDV2 strain showed high titer in VERO-CCL81 cells and no pathogenicity in the piglets. The PEDV2-p103 strain could be evaluated for efficacy in further in vivo immunogenicity and challenge tests.

## Figures and Tables

**Table 1:** Analysis of the deduced amino acids in the Co-26K-equivalent (COE) and SS6 epitopes of the PEDV S proteins.

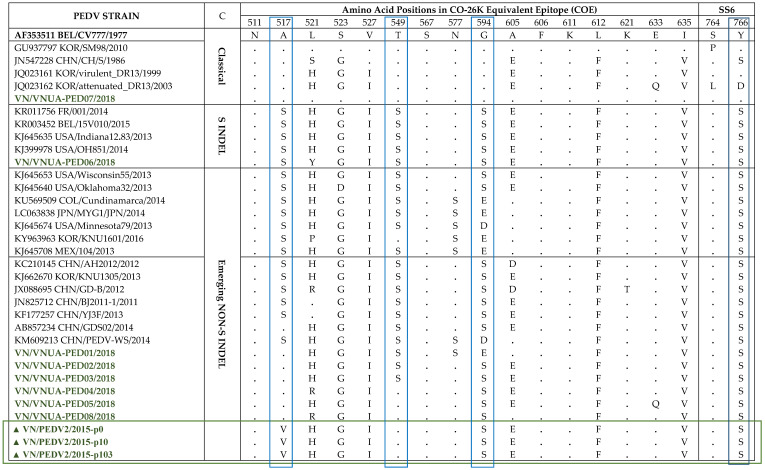

**Table 2:** Summary of mutations in the deduced amino acid of the Spike (S) protein.

	Amino Acid Position												
	27	46	61	62	63	122	193	265	492	647	888	1242	1257
PEDV2-p0	S	V	V	T	C	R	A	H	L	D	G	P	S
PEDV2-p10	.	.	.	.	.	.	.	.	.	.	.	.	.
PEDV2-p26	L	A	.	.	.	.	S	D	V	.	R	S	S
PEDV2-p46	L	A	A	-	-	L	S	D	V	G	R	S	S
PEDV2-p103	L	.	A	-	-	L	S	D	.	G	R	S	F
LLC-MK2/PEDV2-p50 *	.	.	.	.	.	.	.	.	.	.	.	.	.

(*) PEDV2 passage 50 in the LLC-MK2 cell line; “-”, deletion; “.”, amino acids identical to those in the first line.

**Table 3:** Summary of mutations in the ORF3 gene and deduced amino acids of the ORF3 protein.

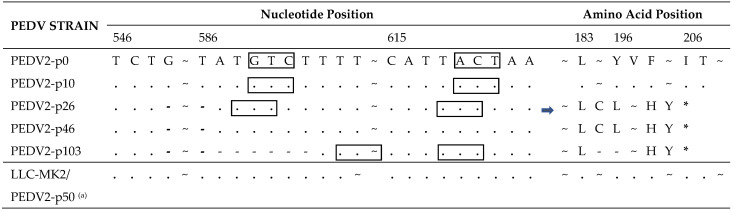

^(a)^ PEDV2 passage 50 in the LLC-MK2 cell line; “-”, deletion; “.”, nucleotides/amino acids identical to those in the first line; “*”, stop codon of amino acid; the squares indicate nucleotide deletions resulting in amino acid mutations.

**Table 4:** Permissiveness of the tested cell lines to PEDV2.

Cell Line	Origin of Cell	PEDV2-p10			PEDV2-p103		
		CPE	ΔCt d0-d4	Log_10_ (TCID_50_/mL)	CPE	ΔCt d0-d4	Log_10_ (TCID_50_/mL)
LLC-MK2	Monkey (*Macaca mulatta*) kidney epithelial	40 h	14.62	6.50	40 h	16.91	6.50
VERO-CCL81	Monkey (*Cercopithecus aethiops*) kidney epithelial	40 h	14.67	6.3	40 h	17.23	6.7
MARC-145	Monkey (*Cercopithecus aethiops*) kidney epithelial	48 h	14.78	6.5	48 h	11.03	5.5
ST	Swine (*Sus scrofa*) testis fibroblast	48 h	12.71	5.9	48 h	14.81	6.1
IPEC-J2	Intestinal porcine (*Sus scrofa*) epithelial cell -J2	No	-	ND	No	-	ND
PK15	Porcine (*Sus scrofa*) kidney epithelial	No	-	ND	No	-	ND

ND, not determined; d, day; h, hour; “-”, negative.

**Table 5:** Primers used for sequencing.

Primer	Sequence (5′-3′)	Strand	PCR Product Size	Reference
ORF3-1	TCCTAGACTTCAACCTTACG	Sense	830	[46]
ORF3-2	GGTGACAAGTGAAGCACAGA	Antisense		
SF1	TCATCCATTAGTGATGTTGT	Sense	1754	[47]
SR1_VN	AAATTGTCTAGTGTCAAC	Antisense		This study
SF2_VN	CCATTCAGCGTATTCTTTATTG	Sense	1693	This study
SR2_VN	ATAGCCTCTTTAACACTCTC	Antisense		
SF3_VN	GATGAAGACTATAAGCGCTG	Sense	1518	This study
SR3_VN	GCTCCAACTCTTGGACAGC	Antisense		

## Data Availability

The data presented in this study are available on request from the corresponding author.
